# The Conservative Treatment of the Dental Pulp When Exposed, Versus Devitalization

**Published:** 1883-11

**Authors:** George Henry


### THE DENTAL REGISTER.

Vol. XXXVIII.] NOVEMBER, 1883. [No. 11.

### COMMUNICATIONS.

### The Conservative Treatment of the Dental Pulp When Exposed versus, Devitalization.

BY MR. GEORGE HENRY, L.D.S.

[Read before the Odontological Society of Great Britain.]

Mr. President and Gentlemen:—As some remarks I made in the discussion at our November meeting may fairly be construed into a protest against the practice of devitalizing dental pulps, based on an abstinence from the use of arsenious acid, or any other destructive escharotic for the purpose of destroying vital pulps, for the last three years; and, observing how many bearings on the question were involved, and that my crude and unprepared observations ill-conveyed my matured convictions on this important subject, I have gladly accepted the invitation of our Secretary, Mr. Turner, to read a brief communication defining my views, deeming it at once a privilege, a pleasant duty to the society, and a satisfaction to myself.

At the outset I must crave indulgence, for I have little that is new to present for consideration, unless it be a certain success in carrying out an old cherished principle—"the conservative treatment of the pulp in health and disease;" and this—unlike most of the contributions I have met with on the subject—without reserving any justification for the practice of destroying the tooth pulp.

I purpose first to enumerate some reasons for our best efforts in preserving the dental pulp; secondly, to express objections to the practice of devitalizing pulps, with stress upon the use of arsenious acid, the most approved agent for the purpose. I shall then describe concisely my treatment of exposed pulps, be they healthy or suppurating, with typical cases, and conclude with kindred observations, commending the matter to your consideration for what it is worth.

It will be no part of my subject to trace the progressive pathological steps towards "exposure," nor to treat on the physiological characteristics of the pulp. These have been so graphically portrayed in our standard dental works and in previous papers read before the society, that I may, without further introduction, proceed as indicated.

Why should we aim at the conservation of the dental pulp? Of first importance is the fact that its functions are not completed with its elaboration of the dentine, but they are continuous through life. The pulp, the chief source of nutrition to the tooth, has an intimate structural connection with the dentine by means of multitudinous nerve-filaments radiating from the odontoblast layer, and traversing the entire body of the tooth; destroy this connection, and we sacrifice the vitality of the dentine. The healthy pulp, by virtue of its dormant developmental power, resists the intrusion of caries, or, to quote from Mr. Tomes' "Manual," "The stimulus or irritation transmitted to the pulp starts afresh the process of calcification in that organ;" and by this same process, the pulp cavity of sound teeth in advancing life becomes almost obliterated. The building up of a tooth, is therefore, a lifelong process, and an all-wise purpose is frustrated by the needless destruction of this central power. Moreover, the presence of the pulp governs the healthful appearance and color of a tooth; and who amongst us has not heard the expression in reference to a tooth deprived of the pulp—"It feels like a dead tooth?" Its looking like one is too often palpable, as must be acknowledged by all; and this is doubtless due to the disorganized contents of the dentinal tubuli, notwithstanding careful root-filling.

I advance these elementary reasons for preserving the pulp—though they must be familiar to all—simply to support my individual effort in preserving diseased pulps, and to stimulate to a greater sense of the responsibility devolving on the dental surgeon to exercise his utmost skill and patience in preserving so valuable an organ when threatened by disease, and in rescuing it when attacked; for truly the dental pulp is as the *medulla* to a bone, or the pith to a tree.

But, if the practice of devitalizing pulps can fairly be supported by results, what is the particular pathological condition which renders such treatment necessary or compulsory? I admit it has hitherto been the last alternative of those who treat the teeth in a truly conservative spirit; but are there not many who regard the smallest exposure as a warrant for resorting to destructive escharotics—aye, who even expose a pulp, with the intention of its more certain destruction, and others who, confronted by an irritable pulp, for lack of knowledge how better to treat it, consign the entire pulp to destruction?

We have only to turn to the discussion at our meeting in November last to see that the advocates for the use of arsenious acid for destroying the pulp are far from unanimous as to the pathological condition demanding such treatment; and, in quoting the opinions of gentlemen justly eminent, I trust my motive will not be misunderstood. For instance, Mr. Kirby "never applied arsenic without cutting off a portion of the surface of the pulp with a small spoon excavator." This vital condition of the pulp, admitting of such excision, to my mind implies one amenable to conservative treatment. At any rate, the treatment I am about to advocate would undoubtedly save such a pulp. One of the earliest pioneers of the conservative treatment of the pulp—by capping this organ—Mr. Thomas A. Rogers,* still holds the opinion "that when suppuration of any part of the pulp had been set up, it was necessary to destroy the whole pulp." But, as far as I am concerned, this suppuration of the pulp has long ceased to be a formidable barrier to its conservation, as I hope presently to show in typical cases.

Mr. Coleman "employed arsenic as an antiseptic in certain dif-

* *Vide* "Transactions of the Odontological Society," 1856-57, vol. I.

ficult cases where only a small portion of dead pulp remained in the roots," such condition in his estimation meriting conservative treatment; but I cannot help thinking most operators would prefer to remove such dead remains by means of a barbed extirpator and syringing, rather than illustrate an undoubted antiseptic property in arsenic, it having the more serious and important property of destroying vitality, and its fluidifying action and consequent risk of spreading through the apical foramen of a tooth, rendering its use dangerous, especially in young persons. Through the postponement of my paper, I have had the pleasure of reading that of Mr. Coleman in the "Transactions" for December last, and the case he cites in reply to a question as to the preference for his treatment with arsenic to that of extirpating dead pulps and root-filling, has certainly surprised me; his conservative efforts having reached the point of preserving absolutely necrosed teeth—so far decomposed, that a slice of the tooth itself would be found to be in a putrid condition.* I have always held the extraction of such teeth to be sound practice, and with due deference to Mr. Coleman's great experience, I still fail to realize that the power of arsenic to arrest decay and putrefaction in tissues can be advantageously used in connection with the teeth. My own treatment presented another phase in the November discussion, and was intendend to represent total abstinence from the practice of devitalizing dental pulps.

But, it was asked of those gentlemen who opposed the use of arsenic, "What evil results had followed its use in their experience?" Of course, looking at the destruction of the pulp as an evil, I object to the use of arsenious acid for this avowed purpose. In the next place, considerable suffering often attends the action of arsenious acid on the pulp; and, to show the uncertainty in this respect, I may quote from Mr. Woodhouse's valuable paper on the treatment of the pulp,† in which he says, "If the pulp has decidedly begun to waste, you may promise your patient very little suffering; but, if it is nearly perfect and healthy, it is safer to make no such promises." Again, have not many pulps

* *Vide* "Transastions of the Odontological Society," vol. i., p. 70.

† "Transactions of the Odontological Society," vol. v., New Series, p. 205.

been unintentionally destroyed through the application of arsenic for obtunding sensitive dentine, so that now the generally accepted course is to reject its use entirely for this purpose. Has not defective manipulation in applying this powerful irritant poison and fluidifying caustic to exposed pulps frequently been followed by painful and serious consequences through its accidental contact with the gums and adjacent structures? Is there not abundant testimony to the fact, that consequent on the destruction of the pulp with arsenic the value and durability of a tooth have been greatly limited? Nor can we ignore the extreme difficulty in some instances of removing all disorganized remains, which, when left behind, incur the danger or annoyance of chronic gum-boils, which has been designated "safety-valves," but are really "vents" of an offensive and objectionable character. I cannot refrain—at this point—from quoting from a most interesting paper by Mr. Underwood—the first I ever heard read on the treatment of the pulp, in April, 1857.* Referring to the immediate and unmistakable effect of arsenic, Mr. Underwood says, "The tooth which but yesterday was the seat of the most excruciating pain, is to-day literally a dead substance in the mouth; but, if we wait a day or two after the tooth is plugged, we find that the periosteum which covers the roots of the teeth is affected." Further on he says, "I do not go the length of some writers, who lay it down as an axiom, that we must on no account use this agent; but I do say, that in the great majority of cases where it is employed, in a few months' time we have periostitis, the plug has to be removed, and in all probability ere long the tooth shares the same fate." I must, however, content myself with referring those gentlemen specially interested in contemplating the dark side of arsenic, to the paper itself.

In connection with this part of my subject, one point—on which I think all are agreed—seems to assert itself, and should act as an additional stimulus to conservative effort, that is, the fact that cases of complete death of the pulp are the most treacherous to treat; and I hope to win others to consider that

* *Vide* "The Quarterly Journal of Dental Science," vol, i. p. 101.

disorganized pulps presented for treatment, through the timid procrastination and long-suffering of patients, are numerous enough, without our voluntarily adding to the number.

Presuming sufficient evidence has been adduced to show the desirability of avoiding the use of arsenic in our treatment of the living pulp, I hasten on to describe my treatment; prefacing this with a few words as to the most suitable material for capping or protecting exposed pulps, and the chemical agent best adapted for restoring and preserving the same, in my experience.

After trying most of the materials recommended for protecting the pulp, I have come to the conclusion that pink bibulous paper answers our purpose better than anything else, for the following reasons: It is non-metallic; its softness and flexibility, when moistened, enables us to adapt it with precision; its absorbent property serves to retain an approved antiseptic application; it is easily placed *in situ*, and the pink color is a help to correct adjustment in difficult situations.

Other materials may doubtless be employed with good results, but anything of a stiff nature, or that cannot be accurately adjusted, I believe must necessarily be an imperfect protector, and that bridging over a pulp is a mistake, since the slightest air-vacuum or atmospheric contact is favorable to septic influence, and therefore a hindrance to success. For this reason I cannot agree with Mr. Hutchinson's plan of burnishing the leaden cap he employs into a saucer shape, with the object of allowing the force to be spent on the lead and not on the nerve, as this is calculated to press in a minute quantity of air, sufficient to endanger permanent success; besides which, in my experience, anything metallic as a protector in contact with the pulp is contra-indicated.

With regard to the chemical agent most valuable to us at once as a coagulating caustic and a powerful non-irriating antiseptic, having tried nitric acid, chloride of zinc, nitrate of silver, creosote, and carbolic acid, I find the last named alone meets all our requirements without drawback.

Nitric acid has proved, in my hands, too corrosive and difficult of application to an exposed pulp, without running the risk of undermining the stability of the adjacent dentine. Chloride of zinc produces intense pain and suffering when applied to an exposed pulp; at the same time, the free acid in the oxychloride is valuable in obtunding the sensitiveness of the dentine around the capping employed, without risking the loss of vitality beyond, as does arsenious acid. Nitrate of silver stains the teeth. Creosote has its unpleasant side, and is less valuable than the purer preparation of carbolic acid. I have tried the pepsine paste and salicylic acid for the sake of their antiseptic property, but my success with carbolic acid has been so uniformly satisfactory, that I look upon it as the most valuable agent we can employ; believing that it converts the suppurating surface of the pulp into a healthy one, and promotes a normal action beneath the blanched film; how else can we account for the permanent comfort secured to teeth correctly treated with it? No doubt an explanation is to be found in the different action of carbolic acid "under cover," and when under atmospheric influence.

I believe this to be a most important and interesting subject, demanding closer investigation; our knowledge as to the nature of the white film produced by corbolic acid, and its eventual changes "under cover" being far too obscure. We do not appear to get a true eschar in the sense of that produced by nitrate of silver or nitric acid; there is no dry slough, crust, or scab, which separates from the healthy tissue; and I would draw attention to the fact that we often see the mucous membrane accidentally blanched when applying carbolic acid to teeth, an appearance identical with that produced with the ether spray, and in neither case is there any slough or destruction of the surface, but in a few minutes the part resumes its normal appearance. In each, there would appear to be a temporary coagulation of the albuminous or fibrinous element in the part affected. The question arises: Is the theory of an insoluble layer correct? I personally believe that we have a valuable temporary coagulation in the one case equivalent to the congelation in the other—both subsiding gradually under healthy vital action. But I can only thus advert to a subject, which I trust will soon meet with clearer elucidation at the hands of some more competent member of the society.

In order to render this paper complete, I must detail my treatment in an ordinary case of exposure, and by exposure I mean a pulp so actually visible and uncovered, either by accident or the intrusion of caries, as to be under the septic influence of decaying matter, or the fluids and atmosphere of the mouth. To be brief, we will suppose that in preparing a cavity on the mesial surface of the first lower molar, the pulp has been needlessly but accidentally exposed, and perhaps punctured. If pain be occasioned, this is readily alleviated with one of the favorite anodynes, such as aconite and chloroform, camphorated chloroform, or, better still, carbolic acid. The cavity finally prepared, a small pledget of wool charged with carbolic acid is kept in contact for from five to ten minutes, according to the extent of the exposure; for if the puncture is only sufficient to cause a slight bleeding, it will not be necessary to bare the pulp, but if a visible exposure has to be dealt with, this should be positively blanched, and so prepared for innocent contact with a protecting layer. This layer, consisting of a small circular or oval bibulous pad, moistened with carbolic acid, is carefully adjusted in juxtaposition with the blanched pulp, overlapping the aperture about half a line or more. This done, a few seconds suffice to mix the osteo, arranged ready to hand on a glass palette. Insert the same without undue pressure, hollow it out, and trim the edges, undercutting for the permanent metallic plug, which may be inserted as soon as the osteo has firmly set.

When disease has accomplished the work of a simple exposure, the treatment will be almost identical, apart from the previous existence of toothache, which would involve temporizing. In such a case the odontalgia would be relieved locally by a selected anodyne dressing protected with mastic, the gums painted with iodine and aconite, and in inflammatory cases an aperient prescribed.

Thus far, many are accustomed to practice almost the same operation under similar circumstances. But, I ask, has this plan been consistently carried out with diseased pulps, to which I consider I have proved it to be applicable in practice?

I will now describe my treatment of suppurating pulp; and it is satisfactory to know " that the supperative process"—I quote from Wedl's "Pathology"*—is almost always developed as a sequence to caries, and is limited to the superficial layer of the body of the pulp or of the root portion corresponding to the carious locality." This condition ascertained, I remove all trace of decay, and then syringe the suppurating surface of the pulp with carbolized warm water, which soon reveals to what extent the pulp has suffered. The next point is perhaps the most important that I have to urge, since to its omission may, I believe, be traced the numerous failures which are deplored by all who have earnestly desired to preserve such pulps, but have had to seek refuge in devitalization. This next step is to cut down the surrounding walls of dentine, so as to be on a level with the surface of the pulp, which may in all cases be accomplished with suitable sharp spoon excavators, and that invaluable aid, the burring engine, so securing the direct apposition of a temporary carbolic acid dressing. This I have rarely to renew more than twice, at intervals of a few days, governed by the perfection of the previous treatment, and when the tooth does not admit of being stopped at the second interview, I protect the fresh dressing with mastic. The suppurating surface having been changed to a healthy one, I proceed to apply the bibulous layer, securing a strict adaptation in contact with the blanched pulp, when the operation is completed by filling temporarily with osteo, or lining the cavity with this materal and inserting a good amalgam filling; or if a gold plug be contemplated, it is wiser to fill with osteo and defer the gold filling for a reasonable time.

It is scarcely needful to add that freedom from tenderness on external pressure—that is, from periodontal mischief or neuralgic irritation—must be ensured previous to plugging.

Difficult situations may be met by freely filing away the tooth; and I am bound to contend from experience—without wishing to lay down a hard-and-fast rule, each case presenting its peculiar features—that the above treatment, with certain modifications, meets all cases of exposed dental pulp, be they healthy, irritated, inflamed, or suppurating, when this organ has not become irrepa-

* "The Pathology of the Teeth," by Carl Wedl, p. 179.

rably gangrenous or dwindled to the condition of dead matter only to be met by extirpation and root-filling; and I think this treatment, carefully carried out, commends itself for:

1. Simplicity and painlessness.
2. Time saving.
3. A general absence of supervening symptoms.
4. Its wide applicability to all cases of exposed vital dental pulp; and,
5. Its *rationale*, taking the peculiar *habitat* of the pulp into consideration, is sufficiently analogous to the surgical treatment of other lesions of the body.

A few typical cases are appended to this paper, and I shall try your patience but a short time longer, whilst offering a few concluding observations.

Constitutional treatment must depend on the presence of systemic disturbance, which does not of necessity accompany the local mischief, and when it does it is commonly *sympathetic*. With this impression I may incur the charge accredited to most writers on this subject, of being too much engrossed with the "local." Still, I am persuaded that topical treatment stands first in the way of removing local irritants, and that we have a right to count much upon the recuperative power of the pulp, that *vis medicatrix naturæ* which befriends us in the treatment of other lesions of the body, from cuts, splinters under the skin, burns, etc. The peculiar diathesis of the patient may favor or retard the progress of healing, and in so far the local treatment may be seconded by judicious antiphlogistic remedies, aperients, and astringent lotions. Constitutional depression of vital power or exhaustion after illnesses simply points to the necessity for temporary local expedients; but the principle of the local treatment advocated must not be departed from.

I desire to give emphasis to one feature in the treatment, and that is, whatever the state of an exposed pulp, short of gangrene, *cut down to it*, or level the adjacent dentine with the pulp's surface. I am convinced we cannot treat it effectually so long as a space exists between the pulp and the aperture leading to it; a fact which has more than once been impressed upon my mind through a carbolic acid dressing failing to blanch the pulp ; showing that the caustic had not touched it, as the inevitable result of contact is a white film or eschar. It is at this point that most operators stop short in their conservative treatment of diseased pulps. Mr. Woodhouse says in his paper already referred to, "When I ascertain that the pulp appears shrunk into the cavity, I at once decide to destroy it, as I consider it a sure sign that its vitality has been lowered, and that it would therefore perish under conservative treatment." A deduction which I believe to be erroneous.

If the pulp be inflammatory, it may with advantage be made to bleed, so relieving the hyperæmic condition, after which gentle syringing with carbolized warm water will have a beneficial effect, and prepare it for a temporary dressing.

When an aching tooth is not relieved in reasonable time by anodyne treatment, I have frequently found that exposing the pulp is effectual in affording relief, and in some instances suppuration will be found to have commenced, this morbid process under compression giving rise to aggravated pain.

I think the happy results attending the above treatment tend to show that the local destruction of the odontoblast layer does not prevent ossification of the pulp; but the greater our success the less our opportunity of gaining information as to the actual physical changes in the pulp so treated. Time will undoubtedly clear up the difficulty when such teeth, from remote causes, may come back to us.

Failures may generally be traced to one of two causes: defective manipulation in difficult situations, or the presence of free dentine bodies in the pulp, at once so difficult to diagnose, and so prolific of neuralgic irritation.* When we are exceptionally

---

* Soon after contributing a paper on the subject of "Secondary Dentine and Free Dentine Bodies as they affect the Preservation of the Pulp," to the British Journal of Dental Science, for April, 1872, a most interesting case of this kind occurred in my practice. A lady resident in St. Leonards had suffered periodically from the most troublesome neuralgia, and had associated this with an upper second molar, right side. The tooth had been ably plugged with gold in Manchester ; unstopped and refilled by a much respected practitioner in London; and this annoying ordeal was again submitted to at my hands, perfect comfort having been enjoyed for ten days before I ventured to stop the tooth with white enamel. I should baffled, and untoward symptoms succeed our efforts to save a pulp, we have an alternative in the operation of rhizodontryphy, and I, amongst others, set a high value on this expedient; but it is only when the first warning of irritation in a stopped tooth has been too long neglected that drilling a vent divulges a dead pulp, and an irritated pulp may be thus relieved without necessarily leading to death.

Fifteen years ago arsenious acid was looked upon in America as the most important article in the dental pharmacopœia, because it enabled the dentist to achieve far more in conservative dentistry than any other one thing. But this so-called "conservative dentistry" meant the preservation of a tooth minus the pulp—"the shell without the kernel." I trust the dental student of to-day is imbibing a truer perception of what should be paramount in preserving the teeth—I mean the conservation of the dental pulp.

In conclusion, let me quote a conservative testimony from the far North: Dr. Brazier, of Stockholm, said at a discussion on this subject at Hamburg in August last, "He never distroyed a pulp. The day had gone by when arsenic should be used, unless a dentist wished to cultivate a batch of abscesses." No doubt many, East and West, are working in the same direction.

I am deeply conscious of the imperfect manner in which this conservative plea for diseased dental pulps has been raised, and beg to disclaim any controversial intention in my method of advocating—not a mere theory—but what has been put to practical test for the last three years, with results which encourage me to pursue the same line of treatment. This treatment, aided by diagrams, I have now sought to reduce to a simple definite method, which I would earnestly commend for trial by all who have hitherto failed in their endeavors to restore and preserve diseased pulps. A sense of duty alone has impelled me to respond to a feeling which has always existed in the society, that individual members should give their personal experience.

---

mention that caries had not reached the nerve, and the cavity, easy of access, was on the buccal surface. A fortnight only elapsed when the patient returned, bent upon having the tooth extracted. This done, I split the tooth open with a vice, and, occupying the upper part of the chamber of the pulp, were found two large independent nodules of secondary dentine.

